# Usefulness of somatosensory-evoked potentials for monitoring cerebral perfusion during transcarotid transcatheter aortic valve replacement: a case report

**DOI:** 10.1186/s44215-025-00219-0

**Published:** 2025-08-18

**Authors:** Koji Okamoto, Yuma Motomatsu, Meikun Kano, Kyohei Meno, Yujiro Ura, Takahiro Mori, Kisho Otani, Shujiro Inoue, Hiromichi Sonoda, Akira Shiose

**Affiliations:** 1 Department of Cardiovascular Surgery, National Hospital Organization Kyushu Medical Center, 1-8-1, Jigyohama, Chuoku, Fukuoka, 810-0065 Japan; 2 Department of Cardiology, National Hospital Organization Kyushu Medical Center, Fukuoka, Japan; 3 Department of Cardiovascular Surgery, Kyushu University Hospital, Fukuoka, Japan

**Keywords:** Transcarotid transcatheter aortic valve replacement, Somatosensory-evoked potentials, Intraoperative monitoring of cerebral ischemia

## Abstract

**Background:**

Transcarotid transcatheter aortic valve replacement (TAVR) is a safe procedure with a low incidence of cerebral infarction and has recently become the first-choice alternative approach. This procedure requires temporary occlusion of the common carotid artery (CCA). CCA clamping during surgery may help reduce the risk of embolism caused by debris; however, the risk of hemodynamic stroke cannot be entirely ruled out. Therefore, intraoperative monitoring of cerebral ischemia is essential. Regional oxygen saturation (rSO_2_) monitoring is commonly used, but can only measure local mixed venous oxygen saturation in the frontal lobes. During carotid endarterectomy (CEA), a combination of multiple monitoring methods for intraoperative cerebral ischemia is recommended. Similarly, we used somatosensory-evoked potentials (SEPs) in conjunction with rSO_2_ monitoring.

**Case presentation:**

A 92-year-old male patient with a history of dyspnea on exertion was diagnosed with severe aortic valve stenosis (AS) using transthoracic echocardiography (TTE). Contrast-enhanced computed tomography (CT) revealed a shaggy aorta extending from the aortic arch to the descending aorta. Preoperative magnetic resonance angiography (MRA) of the head showed slight narrowing of the anterior communicating artery. Considering the patient’s age, frailty, and vascular pathology, we performed transcarotid TAVR while monitoring rSO_2_ and SEPs for intraoperative cerebral ischemia. No significant decreases in rSO_2_ values or SEPs amplitudes due to occlusion of the left CCA. The procedure was successful, with no postoperative stroke, and the patient had an uneventful recovery.

**Conclusions:**

In transcarotid TAVR requiring CCA occlusion, monitoring cerebral ischemia with both rSO_2_ and SEPs may help prevent perioperative hemodynamic cerebral infarction.

## Background

TAVR is a reliable technique with excellent outcomes for the treatment of aortic valve stenosis (AS). In 2019, femoral access increased to 95.3% and remains the first-choice approach for a less invasive procedure [1]. However, some patients require an alternative access route due to anatomical limitations.

Transcarotid transcatheter aortic valve replacement (TAVR) has been established as a safe alternative, and is increasingly chosen in clinical practice [1–5]. Transcarotid TAVR is considered less invasive than transapical TAVR and transaortic TAVR, and has been reported to be associated with a lower incidence of stroke compared to transsubclavian TAVR [3, 4]. Carotid artery occlusion during the procedure may reduce embolic risk but does not eliminate concerns about hemodynamic stroke. Therefore, intraoperative monitoring of cerebral ischemia is essential. Regional oxygen saturation (rSO_2_) monitoring is commonly used because it is easy to use, non-invasive, and portable. However, it can only measure local mixed venous oxygen saturation in the frontal lobes and has a poor positive predictive value and low specificity. Similarly, in carotid endarterectomy (CEA) requiring carotid artery occlusion, multiple intraoperative cerebral blood flow monitoring techniques are widely used [6–9]. Therefore, in this case, somatosensory-evoked potentials (SEPs) monitoring was performed in addition to rSO_2_ monitoring.

## Case presentation

**Fig. 1:**
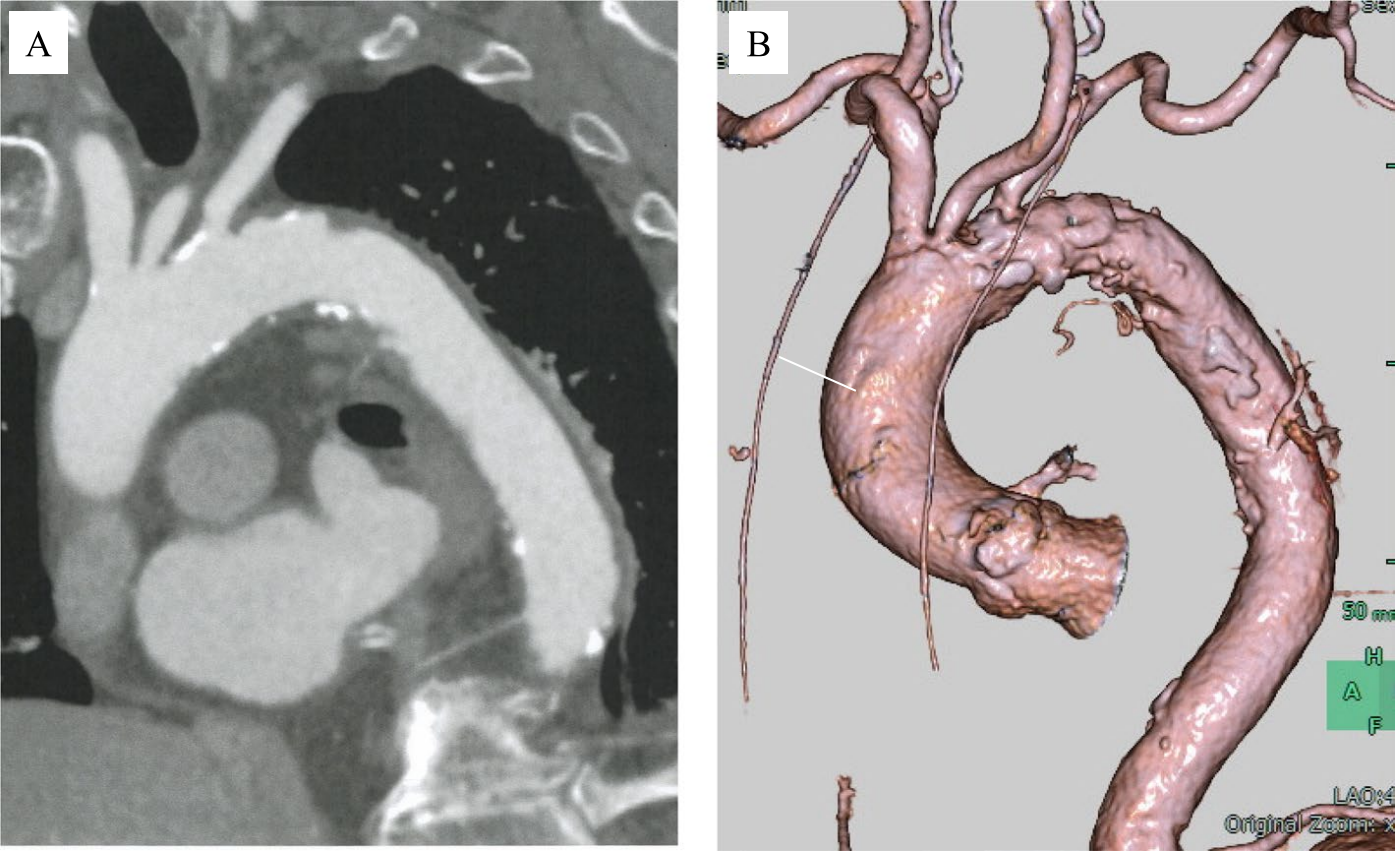
Preoperative CT. **A** A shaggy aorta from the aortic arch to the descending aorta. **B** Significant tortuosity of the right subclavian artery and severe calcification of the left subclavian artery

**Fig. 2:**
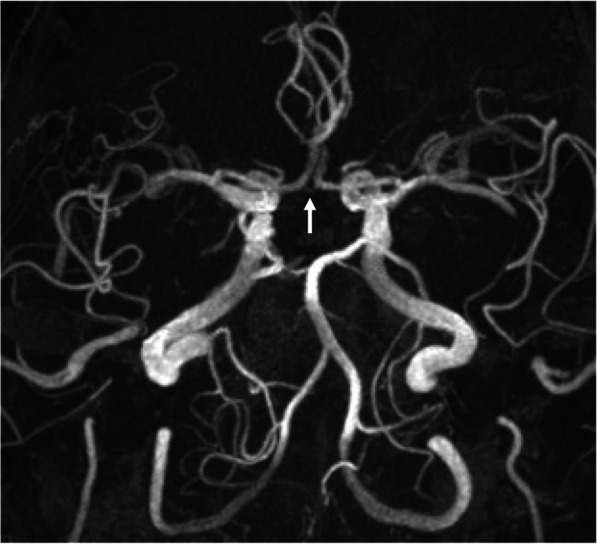
Preoperative head MRA. The anterior communicating artery is slightly narrowed

**Fig. 3:**
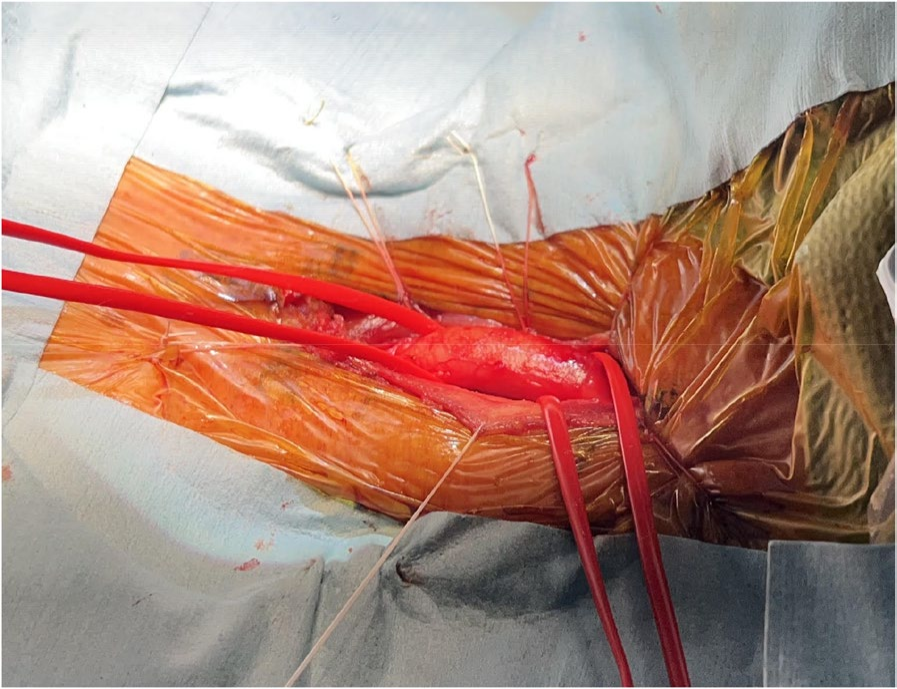
Intraoperative finding of the left CCA

**Fig. 4:**
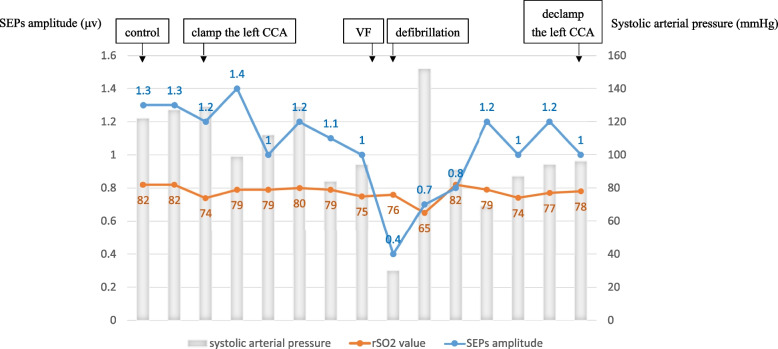
Intraoperative changes in rSO_2_ value and SEPs amplitude

**Fig. 5:**
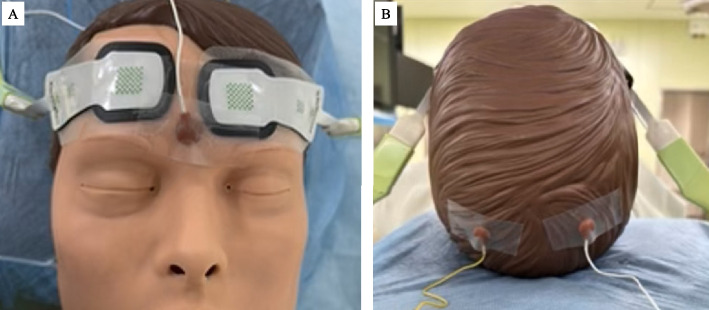
A photograph illustrating the placement of rSO_2_ patches and SEP electrodes on a model. **A** Locations for rSO_2_ sensor attachment. **B** Locations for SEP electrode attachment

A 92-year-old male patient was diagnosed with severe AS using transthoracic echocardiography (TTE). The Society of Thoracic Surgery score was 9.9%. Given the patient’s age and frailty, TAVR was performed. Contrast-enhanced CT revealed a shaggy aorta extending from the aortic arch to the descending aorta, with significant tortuosity of the right brachiocephalic artery and severe calcification of the left subclavian artery (Fig. 1A, B). Therefore, we considered the femoral and subclavian artery approaches unsuitable. Head magnetic resonance angiography (MRA) revealed an intact Circle of Willis with slight narrowing of the anterior communicating artery (Fig. 2). Since carotid artery occlusion could not be ruled out as a cause of ipsilateral cerebral ischemia, we considered multiple intraoperative cerebral blood flow monitoring methods necessary. We used SEPs monitoring in addition to rSO_2_ monitoring, and a femoro-carotid shunt was planned if cerebral ischemia was detected. SEPs amplitude was measured every 5 min. Under general anesthesia, the left common carotid artery (CCA) was surgically exposed through a 4-cm vertical incision (Fig. 3). After clamping the distal left CCA to prevent atheroma migration, a 6-Fr short sheath was inserted. No significant decreases in rSO_2_ or SEPs amplitudes were observed due to occlusion of the left CCA (Fig. 4). The 6-Fr sheath was removed, and a 14-Fr E-sheath was advanced over the guidewire and into the aortic arch. A Sapien 3 Ultra RESILIA 26 mm valve (Edwards Lifesciences, Inc., Irvine, CA, USA) was successfully deployed under rapid pacing. Ventricular fibrillation (VF) occurred after rapid pacing was discontinued, requiring immediate defibrillation. SEPs amplitude significantly decreased only during VF, but recovered after defibrillation and restoration of sinus rhythm (Fig. 4). The total occlusion time of the left CCA was 65 min due to the complexity of the procedure, influenced by severe aortic angulation, often referred to as the “horizontal aorta.” Despite the prolonged occlusion time of the left CCA, the procedure was safely completed without complications under rSO_2_ and SEPs monitoring (Fig. 5). Intraoperative systolic blood pressure was tried to control within the range of 100 to 140 mmHg with the use of vasopressors such as phenylephrine or norepinephrine. The patient’s postoperative course was favorable. TTE on postoperative day four revealed good artificial valve function with trace paravalvular leakage. The patient was discharged on the seventh postoperative day.

## Discussion

Transcarotid TAVR has a low risk of cerebral infarction among alternative TAVR approaches [2–5]. However, four potential stroke mechanisms have been identified during transcarotid TAVR: (1) embolism from arterial puncture; (2) access site trauma leading to thrombosis; (3) inadequate collateral perfusion; and (4) embolization of debris from the aortic valve [5]. Recent evidence suggest that the absence or dysfunction of collateral segments within the Circle of Willis may increase the risk of perioperative cerebral infarction in patients with symptomatic carotid stenosis [10, 11]. Assessing the presence of severe calcification or plaques in the ipsilateral CCA, significant stenosis in the contralateral CCA, and the condition of the Circle of Willis preoperatively is essential. In this case, preoperative head MRA revealed slight narrowing of the anterior communicating artery; therefore, intraoperative cerebral ischemia monitoring was deemed essential. Common neuromonitoring methods for assessing cerebral perfusion risk include rSO_2_ monitoring, SEPs, stump pressure, electroencephalography, and transcranial Doppler [6–8]. However, none provides 100% sensitivity and specificity for detecting critical cerebral perfusion during carotid artery clamping under general anesthesia. Therefore, multimodal intraoperative neurophysiological monitoring is recommended [6–8]. Since SEPs and rSO_2_ monitoring are effective in detecting cerebral ischemia in the anterior and middle cerebral artery territories, they may be particularly useful in cases with poor collateral circulation within the Circle of Willis or severe stenosis of the contralateral carotid artery.. According to previous reports, in CEA, an rSO_2_ decrease of more than 20% or an SEPs amplitude reduction of more than 50% is considered an indication for the use of an intraluminal shunt [7, 8]. In this case, although the rSO_2_ value decreased mildly from 82 to 74% due to left CCA occlusion, SEPs amplitude remained stable, changing only slightly from 1.3 to 1.2 μV. Therefore, we concluded that proceeding with surgery was safe. SEPs amplitude significantly decreased to 0.4 μV during VF, suggesting that SEPs may be a more sensitive indicator of cerebral ischemia. If the neurophysiological monitoring values indicate ischemia when the CCA is clamped, we first confirm that the changes are persistent and not due to artifacts. SEPs amplitude is monitored over several stimulation runs to assess whether the decrease is sustained. We then attempt to elevate systemic blood pressure using vasopressors, targeting a systolic pressure greater than 140 mmHg. If feasible, we aim to shorten the carotid clamping time, particularly during the device deployment phase. If neuromonitoring values do not recover within 3–5 min despite these interventions, distal perfusion beyond the clamp site is planned using a 6-Fr sheath placed in the left femoral artery, as described in this report [12]. Routine shunting during CEA has been associated with a high stroke rate than near-infrared spectroscopy-guided selective shunting, possibly due to perioperative embolism during shunt placement or the mobilization of vulnerable plaques within the vessel [13]. Therefore, we believe that a femorocarotid shunt should not be used routinely but only when cerebral ischemia is detected through cerebral perfusion monitoring.

## Conclusion

When performing TC-TAVR requiring CCA occlusion, multimodal intraoperative neurophysiological monitoring with both rSO_2_ and SEPs may enable the rapid identification of stroke-related risks, potentially preventing perioperative hemodynamic cerebral infarction.

## Data Availability

Not applicable.

## Abbreviations

TAVR: Transcarotid transcatheter aortic valve replacement
CCA: Common carotid artery
rSO_2_: Regional saturation of oxygen
CEA: Carotid endarterectomy
SEPs: Somatosensory evoked potentials
AS: Aortic valve stenosis
TTE: Transthoracic echocardiography
CT: Computed tomography
MRA: Magnetic resonance angiography
